# *Mycobacterium shimoidei*, a rare non‐tuberculous mycobacteria pathogen identified by matrix‐assisted laser desorption/ionization time‐of‐flight mass spectrometry

**DOI:** 10.1002/rcr2.428

**Published:** 2019-04-17

**Authors:** Hiroaki Nagano

**Affiliations:** ^1^ Department of Respiratory Medicine Okinawa Chubu Hospital Okinawa Japan

**Keywords:** Cavitary lesion, MALDI‐TOF MS, *Mycobacterium shimoidei*, non‐tuberculous mycobacteria, treatment

## Abstract

Non‐tuberculous mycobacteria (NTM) cause several infectious diseases in humans. This study reports on *Mycobacterium shimoidei* infection in an immunosuppressed 61‐year‐old male with a background of emphysema. His chief complaint was haemoptysis. Chest computed tomography showed a large, thin‐walled cavitary lesion in the upper right lobe. Although NTM were identified in two separate expectorated sputum samples, DNA–DNA hybridization (DDH) failed to identify the species. *M. shimoidei* was finally identified using matrix‐assisted laser desorption/ionization time‐of‐flight mass spectrometry (MALDI‐TOF MS). Following antimicrobial agent susceptibility tests, treatment with clarithromycin, levofloxacin, and ethambutol commenced. Six months post‐treatment, acid‐fast sputum culture was negative and repeat imaging demonstrated improvement of the radiographic abnormalities. This study aimed to assess the utility of MALDI‐TOF MS for successful identification of rare NTM species that are not identifiable by DDH. It is the first report of *M. shimoidei* from Okinawa, which is the only prefecture in Japan categorized as subtropical.

## Introduction

Non‐tuberculous mycobacteria (NTM) are ubiquitous in the environment and can cause various infectious diseases in humans. The prevalence of NTM‐induced lung infections is increasing globally. *Mycobacterium shimoidei* is a slow‐growing NTM that was first isolated in Japan in 1968, gaining species status in 1975 1. Since then, a number of cases of infection have been reported worldwide 1, 2, 3, 4, 5, 6, 7.

## Case Report

**Figure 1:**
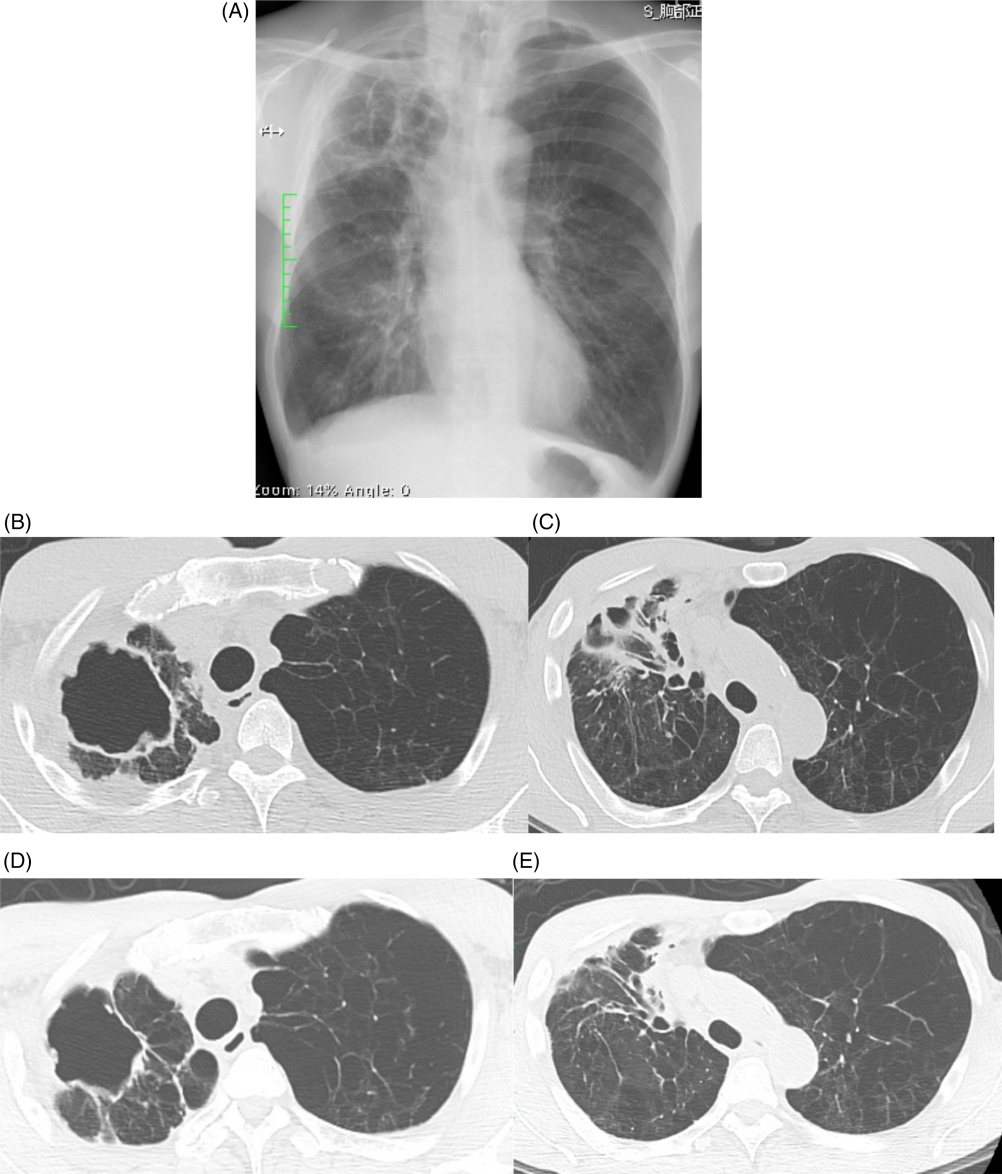
The chest X‐ray on the first visit demonstrated the large cavitary lesion in the right upper lung fields (A). The thoracic computed tomography showed the large, thin‐wall cavity lesion, the thickness of pleural wall, trabecular and linear shadows in the upper right lobe, and bronchiectasis in the upper and middle lobe (B, C). Six months after treatment, the cavitary lesion and trabecular shadows were decreased (D, E).

A 61‐year‐old man from Okinawa, Japan, presented at his local hospital complaining of cough and bloody sputum during the preceding week. He was a current smoker with a history of chronic obstructive pulmonary disease (COPD), and rheumatoid arthritis that had been treated with prednisolone 7.5 mg per day and methotrexate 12 mg per day since 2005 by a primary care physician. He was a professional gardener. Chest X‐ray demonstrated a thin‐walled cavitary lesion in the upper right lobe. He was referred to our hospital for further evaluation. Chest computed tomography (CT) on the first visit showed a large, thin‐walled cavitary lesion, pleural wall thickening, trabecular and linear shadows in the upper right lobe, and bronchiectasis in the upper and middle lobe (Fig. 1A–C). Acid‐fast bacilli were found on Ziehl–Neelsen staining of two separate sputum smears, respiratory specimens were cultured with 2% Ogawa agar, and the resulting bacterial colonies were collected for species identification. Polymerase chain reaction for tuberculosis (TB) was negative. The resulting suspension liquid was tested using a DNA–DNA hybridization (DDH) method from a commercially available identification kit (Kyokuto Pharmaceutical Industrial Co. Ltd., Tokyo, Japan). Finally, an NTM was isolated from two separate expectorated sputum samples by DDH, which failed to identify the NTM species. The patient's haemoptysis symptoms improved spontaneously without treatment, and he was discharged from the hospital on his own judgement. Although we encouraged him to attend regular follow‐ups at the outpatient centre of our hospital, he declined due to personal reasons.

Two years later, in December 2017, the patient was readmitted to our hospital with a recurrence of bloody sputum. The CT scan showed that the cavity in the upper right lobe had extended and the cavity wall had become thinner compared to the previous lesions. Microbiological work‐up again isolated an NTM from two separate expectorated sputum samples, and DDH again failed to identify the bacterial species. The positive cultures were sent to a specialized microbiology laboratory for matrix‐assisted laser desorption/ionization time‐of‐flight mass spectrometry (MALDI‐TOF MS), and *M. shimoidei* was finally identified. The activities of antimicrobial agents were examined by a commercially available drug susceptibility kit for NTM (Kyokuto Pharmaceutical Industrial Co. Ltd., Tokyo, Japan), with the breakpoints derived using *Mycobacterium kansasii* as a reference*. M. shimoidei* proved susceptible to clarithromycin (CAM), ethambutol (EB), streptomycin, amikacin, and levofloxacin (LVFX); and resistant to rifampicin (RFP). After referring to published literature and the advice of an expert on acid‐fast bacteria, the patient was started on a treatment regimen of CAM, EB, and LVFX in January 2018. His haemoptysis decreased gradually and the cavitary lesion improved (Fig. 1D, E). Six months after treatment, acid‐fast culture of sputum was negative.

## Discussion

The purpose of this study is to communicate the potential utility of MALDI‐TOF MS for identification of *M. shimoidei* that is not identifiable by DDH. This is essential for choice of the appropriate antimicrobial treatment. Notably, this is also the first identification of *M. shimoidei* in Okinawa, the southernmost and only prefecture categorized as subtropical in Japan. Species isolated from patients with NTM lung disease are geographically diverse, although the *Mycobacterium avium* complex is the commonest species in most countries, including mainland Japan 8. Recently, our group reported that Okinawa may be one of the few places where the *Mycobacterium abscessus* complex is the predominant pathogen causing NTM lung disease 9.

Previously published details of 25 cases and this case are summarized together in Table 1 1, 2, 3, 4, 5, 6, 7, which is based on the format of the table in the study by Baird et al. 3 Cases with normal chest X‐ray were excluded in Table 1. Previous reports of *M. shimoidei* infections have been predominantly in the lungs, and largely among male patients. The most commonly associated concurrent conditions were COPD, bronchiectasis, and past TB 1, 2, 3, 4, 5, 6, 7. Common symptoms were cough or sputum production, weight loss, dyspnoea, fevers, or sweats. Radiology demonstrated cavitating disease in approximately 70% of patients (Table 1). The infection route for humans is unknown.

**Table 1:** Clinical features, treatments and outcomes of Mycobacterium shimoidei lung infection.

Age (year)/sex	Identfication methods	Symptoms/signs	Other diseases	Radiology	Therapy (time)	Outcome	Reference
56/M	Biochemical identification	Unknown	Not reported	Cavity	Not reported	Died of lung disease	Tsukamura et al. 1
68/M	Bergey's Manual of Systematic Bacteriology	Sputum	TB, Addison's disease	Cavity	INH, SM, RFP, kanamycin (4 months)	Died	Tortoli and Simonetti 7
56/M	Bergey's Manual of Systematic Bacteriology	Sputum	None	Cavity	Unknown	Unknown	Tortoli and Simonetti 7
77/M	Bergey's Manual of Systematic Bacteriology	Sputum	Silicosis	Cavity	INH, RFP, SM	Improved	Tortoli and Simonetti 7
53/F	Genetic assay (16S rRNA)	Fatigue, weight loss	oesophagus cancer	Cavity	INH, RFP, PZA, EB	Died	Mayall et al. 4 1999
68/M	Genetic assays (16S rRNA)	Cough, fever, sputum	TB, gastrc ulcer, pneumothorax, COPD	Consolidation	RFP, EB, PZA, CAM, CPFX (6 months)	Improved	Takayama et al. 5 2006
45/M	Genetic assay (16S rRNA)	Haemoptysis	COPD	Cavity, consolidation	INH, RFP (6 months)	Stable	Saito et al. 2
75/M	Genetic assay (16S rRNA)	Cough, fatigue, fever, weight loss	TB	Cavity, Consolidation	RFP, EB, SM, CAM (12 months)	Improved	Saito et al. 2
53/F	Genetic assays (16S rRNA, etc.)	Cough, haemoptysis	TB	bronchiectasis	EB, AMK, RIF, CAM (18 months)	Improved	Nadia et al. 2013 [13]
83/M	Genetic assays (16S rRNA, etc.)	Sputum	glomerulonephritis	Cavity, Nodules, pleural thicking	CAM, RFP, EB	Improved	Kanaji et al. 2013 6
60/M	Genetic assays (16S rRNA, etc.)	Cough, sputum, weight los	COPD, asthma	Cavities, nodule	Observed	Stable	Baird et al. 3
56/M	Genetic assays (16S rRNA, etc.)	Unknown	Unknown	Unknown	None	Died	Baird et al. 3
75/F	Genetic assays (16S rRNA, etc.)	Cough, sputum, weight loss	COPD, HF, AF, GERD	Cavities, nodules	None	Died of other cause	Baird et al. 3
72/M	Genetic assays (16S rRNA, etc.)	Cough, dyspnoea, weight loss	COPD, bronchiectasis, IHD	Cavity, nodules	Observed	Died of lung disease	Baird et al. 3
62/M	Genetic assays (16S rRNA, etc.)	Cough, weight loss, night sweats	None	Cavity	INH, RFP, PZA, EB (6 months)	Stable	Baird et al. 3
68/M	Genetic assays (16S rRNA, etc.)	Cough, haemoptysis, fatigue	COPD, aspergillus, HTN	Cavities, consolidation	CAM, MFX, SMX (12 months)	Improved	Baird et al. 3
70/M	Genetic assays (16S rRNA, etc.)	Cough, sputum, chest pain	Lung cancer, COPD, bronchiectasis	Cavities	CAM, RIF, EB (12 months)	Died of lung disease	Baird et al. 3
77/F	Genetic assays (16S rRNA, etc.)	Cough, weight loss, fatigue	COPD, GERD	Cavity, nodules	CAM, RFP, EB (18 months)	Improved	Baird et al. 3
68/M	Genetic assays (16S rRNA, etc.)	Cough, sputum, weight loss	COPD, RA, anaemia	Cavity, consolidation	Observed	Stable	Baird et al. 3
76/M	Genetic assays (16S rRNA, etc.)	Dyspnoea, weight loss	COPD, anaemia	Nodules	None	Unknown	Baird et al. 3
84/M	Genetic assays (16S rRNA, etc.)	Cough, sputum, weight loss	Lung cancer, GERD	Mass, effusion	Observed	Died of lung disease	Baird et al. 3
84/M	Genetic assays (16S rRNA, etc.)	Cough, dyspnoea, fatigue	COPD, bronchiectasis	Consolidation	Observed	Improved	Baird et al. 3
29/M	Genetic assays (16S rRNA, etc.)	Cough, dyspnoea, weight loss	CF, bronchiectasis	Nodules	AMK, CFX, AZM, CFZ (24 months)	Improved	Baird et al. 3
74/F	Genetic assays (16S rRNA, etc.)	Cough, sputum	Bronchiectasis	Nodules, consolidation	Observed	Improved	Baird et al. 3
84/F	Genetic assays (16S rRNA, etc.)	Cough, haemoptysis, weight loss	Bronchiectasis, type 2 diabetes, HTN	Nodules	CAM (2 months)	Improved	Baird et al. 3
61/M	MALDI‐TOF MS	Haemoptysis	COPD, RA	Cavity, pleural wall thickening	CAM, EB, LVFX (18 months)	Improved	Nagano 2019 [this case]

Abbreviations: AF, atrial fibrillation; AMK, amikacin; AZM, azithrimycin; CAM, clarithromycin; CF, cystic fibrosis; CFX, cefoxitin; CFZ, clofazimine; COPD, chronic obstructive pulmonary disease; CPFX, ciprofloxacin; EB, ethambutol; GERD, gastro esophageal reflux disease; HF, heart failure; HTN, hypertension; IHD, ischemic heart disease; INH, isoniazid; LVFX, levofloxacin; MFX, moxifloxacin; PZA, pyrazinamide; RA, rheumatoid arthritis; RFP, rifampicin; SM, streptomycin; SMX, sulfamethoxazole; TB, tuberculosis.

MALDI‐TOF MS is a proteomics method that permits rapid and accurate identification of mycobacterium species from positive cultures 10. Whereas only 18 acid‐fast bacteria species have been identified using DDH, MALDI‐TOF MS successfully identified 148 species from NTM isolates 10. Furthermore, compared to other molecular techniques, MALDI‐TOF MS is more cost effective, provides faster identification of mycobacterial isolates to the species level, and often facilitates earlier implementation of more appropriate therapies than is possible with previous bacterial identification methods 10, 11.

Although more information about drug susceptibilities of *M. shimoidei* is required, it is known to be resistant to isoniazid and RFP, and susceptible to EB and rifabutin (RFB) 2, 4, 5. Recently, Baird et al. suggested drug regimens combining RFB, EB, and CAM, with moxifloxacin/LVFX, sulfamethoxazole, pyrazinamide, and clofazimine also being potentially useful 3. In the case presented here, susceptibility testing combined with expert opinion permitted to add LVFX to the treatment regimen for *M. shimoidei*. Increased recognition and understanding of this pathogen are necessary to expedite diagnosis and improve patient outcomes.

### Disclosure Statement

Appropriate written informed consent was obtained for publication of this case report and accompanying images.

## References

[1] Tsukamura M , Shimoide H , and Shaefer WB . 1975 A possible new pathogen of group III mycobacteria. J. Gen. Microbiol. 88:377–380. 1151342 10.1099/00221287-88-2-377

[2] Saito H , Zayasu K , Shigeto E , et al. 2007 Two cases of lung infection due to *Mycobacterium shimoidei*, with special reference to bacteriological investigation. J. Jpn Assoc. Infect. Dis. 81:12–19. 10.11150/kansenshogakuzasshi1970.81.12. 17338311

[3] Baird TM , Carter R , Eather G , et al. 2017 *Mycobacterium shimoidei*, a rare pulmonary pathogen, Queensland, Australia. Emerg. Infect. Dis. 23:1919–1922. 29048290 10.3201/eid2311.170999 PMC5652447

[4] Mayall B , Gurtler V , Irving L , et al. 1999 Identification of *Mycobacterium shimoidei* by molecular techniques: case report and summary of the literature. Int. J. Tuberc. Lung Dis. 3:169–173. 10091886

[5] Galizzi N , Tortoli N , Gori A , et al. 2013 A case of mild pulmonary disease due to *Mycobacterium shimoidei* with a favorable outcome. J. Clin. Microbiol. 51:3467–3468. 23926163 10.1128/JCM.01028-13 PMC3811658

[6] Nobuhiro K , Yoshio K , Shuji B , et al. 2013 Membranous glomerulonephritis associated with *Mycobacterium shimoidei* pulmonary infection. Am. J. Case Rep. 14:543–547. 24367720 10.12659/AJCR.889684 PMC3869631

[7] Tortoli E , and Simonetti MT . 1991 Isolation of *Mycobacterium shimoidei* from a patient with cavitary pulmonary disease. J. Clin. Microbiol. 29:1754–1756. 1761702 10.1128/jcm.29.8.1754-1756.1991 PMC270200

[8] Prevots DR , and Marras TK . 2015 Epidemiology of human pulmonary infection with nontuberculous mycobacteria: a review. Clin. Chest Med. 36:13–34. 25676516 10.1016/j.ccm.2014.10.002 PMC4332564

[9] Nagano H , Kinjo T , Fujita J , et al. 2017 Causative species of nontuberculous mycobacterial lung disease and comparative investigation on clinical features of *Mycobacterium abscessus* complex disease: a retrospective analysis for two major hospitals in a subtropical region of Japan. PLoS One 12:e0186826 10.1371/journal.pone.0186826. 29059250 PMC5653325

[10] Genc GE , Demir M , Yaman G , et al. 2018 Evaluation of MALDI‐TOF MS for identification of nontuberculous mycobacteria isolated from clinical specimens in mycobacteria growth indicator tube medium. New Microbiol. 41:214–219. 29874386

[11] Şamlı A , and İlki A . 2016 Comparison of MALDI‐TOF MS, nucleic acid hybridization and the MPT64 immunochromatographic test for the identification of *M. tuberculosis* and non‐tuberculosis *Mycobacterium* species. New Microbiol. 39:259–263. 27551724

[12] Takayama S , Tominaga S , Tsukada Y , et al. 2006 A case of Mycobacterium shimoidei infection. Kekkaku. 81:537–541. 16972658

